# Is Validating the Cutoff Score on Perinatal Mental Health Mood Screening Instruments, for Women and Men from Different Cultures or Languages, Really Necessary?

**DOI:** 10.3390/ijerph19074011

**Published:** 2022-03-28

**Authors:** Stephen Matthey

**Affiliations:** Department of Clinical and Experimental Sciences, Faculty of Medicine and Surgery, Università degli Studi di Brescia, 25123 Brescia, Italy; travelresearch@hotmail.com

**Keywords:** perinatal, screening, validation, EPDS

## Abstract

*Background*: The most commonly used mood screening instrument in perinatal health is the Edinburgh Depression Scale. The screen-positive cut-off score on this scale, as for others, has been determined, via validation techniques, for over 20 languages/cultures, and for both women and men. While such validation appears to be considered essential, there are studies that could be interpreted to suggest that this is not an important consideration. *Methods*: Selective studies have been chosen to indicate these opposing points of view. *Results*: Examples of studies that support the notion of validating cut-off scores are described, as are examples of studies that appear not to support this point of view. *Conclusions*: (i) Clinical services and researchers need to be mindful of these opposing points of view, and openly discuss them when using screening cut-off scores for their respective populations. (ii) Researchers and Journals need to be more rigorous in ensuring this issue is correctly reported in studies, and/or openly discussed when relevant.

## 1. Introduction

Validation of self-report mood screening questionnaires for the perinatal period, such as the Edinburgh Postnatal Depression Scale (EPDS) [1], is a practice that almost goes without question. Such validation produces an empirically derived cut-off score, or screen-positive score, that guides clinical services to decide when to refer a woman, or man, for further psychological assessment for the presence of whatever mood disorder the scale has been validated against (e.g., major depression; various anxiety disorders etc.). It also provides researchers with the validated cut-off score from which studies investigating issues such as prevalence, risk factors, and treatment effectiveness can be empirically explored.

The usual validation method is to compare the study sample’s scores on the scale against the gold standard of diagnostic disorder status (e.g., DSM or ICD mental health disorders [2,3]), and to calculate its receiver operating characteristics (ROC). These ROC characteristics usually include the scale’s sensitivity, specificity, and its positive predictive value (ppv), at all possible scores on the scale. The best desired mix of these is then chosen (sometimes based upon other resultant statistics) to determine the optimal screen-positive score for that sample, or population from which the sample was drawn.

Thus the EPDS has been translated, and validated, into many different languages, with reported optimum cut-off scores across cultures for depression, or depression and anxiety, currently varying from 4 or more [4] to 19 or more [5]. In addition, the optimal cut-off scores for men have been calculated in several different languages and/or cultures. Examples of such validation studies for women, with their recommended cut-off scores (which may be for different disorders, such as major or minor depression, or anxiety), include those in Ethiopia [6]: 6, 7, or 8 or more; Nigeria [7]: 9 or more; Vietnam [4]: 4 or more; Malta [8]: 14 or more; Denmark [9]: 11 or more, as well as the original English EPDS validation study [1]: 13 or more. For men, examples of such studies, with their recommended cut-off scores, include those in Vietnam [10]: 5 or more; England [11]: 11 or more; Saudi Arabia [12]: 9 or more; Sweden [13]: 12 or more; Australia [14]: 6 or more. and Italy [15]: 13 or more.

Some of the reasons investigators give as to why different optimal cut-off scores were obtained in their sample, compared to other cultural or gender groups, include that such groups may differ in their expression of depression or the actual symptoms experienced [4,9,10,12,16], as well as differences in the comprehension of the screening scale’s items [4,6]. These reasons, as opposed to more procedural or psychometric reasons (e.g., different caseness criteria, or different gold-standard interviews, used across studies), would support the need to conduct validation studies in different populations to ensure any such emotional expressiveness, comprehension, or symptom experience differences are taken into account when screening women, and men, for possible emotional health difficulties.

All such validation studies thus inherently support the belief that the scale must be validated for each specific population, and that the optimal screen cut-off score should then be used in similar populations, both within research and clinical settings. The aim of this opinion piece is to create a critical debate in health professionals involved in the use of such screening scales, by describing studies that appear to support the opposite belief, that such scales do not need to be validated for different populations. This belief is either stated within the studies themselves, or can be inferred when a study has not used the previously validated cut-off score for that population without a reasonable rationale.

The purpose of this paper is therefore to question whether indeed such validations need to be done.

## 2. Materials and Methods

Selective studies will be reported that support the argument that screening scales, specifically the EPDS, should be validated regarding the optimal screen-positive cut-off score for each culture and/or gender (and sometimes for both pre and postnatal periods). Similarly, selective studies will be reported that appear to support, or could be interpreted to support, the counter argument—that there is now no need to validate the EPDS cut-off score for different cultures or genders, as a single cut-off score can be used for people from all cultures and/or genders. As some of what I say below may seem as criticism of various studies, I wish to emphasise that these are being used only as examples, and I do not exclude the fact I too may have made similar errors in my studies.

This methodology, of selectively reporting a number of studies to highlight the points being made, is considered by the author to be appropriate in the context of this being an opinion piece article, not a research study. This methodology has been used previously by the author [17] in a related discussion about emotional health screening with the EPDS.

The studies selected were chosen on the basis that (a) they provided examples which supported either position regarding whether or not validation of emotional screening scales needs to be undertaken across different populations; (b) the author was aware of these studies from his extensive reading of the literature over many years, and (c) sufficient numbers of such studies have been reported in this paper (approximately 22 for each side of the argument) to demonstrate that both sides of the argument are not simply supported by ‘outlier’ type studies (e.g., by just one or two studies).

## 3. Results

### 3.1. Studies Supporting the Validation of the EPDS across Cultures or Genders

The studies cited above, providing the optimal cut-off score on the EPDS for women or men in their specific culture or country, are by their very nature studies which clearly support the view that cross-cultural/gender validation studies need to be conducted if we wish to have empirical evidence guiding both clinical and research practice.

In addition some reviews also provide support for this argument. Gibson et al. [18], in their review of 37 EPDS validation studies across various cultures. concluded that their findings implied that “different cut-off scores should be used in different cultural groups” (p. 359). Kozinsky and Dudas [19], in their review of 11 EPDS validation studies, also concluded this, stating “it is not advisable to use universal cut-off scores (on the EPDS), as there can be cultural differences …” (p. 101).

Housen et al. [20] stated that their study, validating various mental health instruments in India, showed “the importance of culturally adapting and validating screening instruments” (p. 361), while Heck et al. [21] commented that the EPDS may be culturally biased, and that its items need to be validated for conceptual equivalence in women from culturally diverse backgrounds.

Tran et al. [4], in studying the validity of the EPDS and other mood instruments for women in Vietnam (and on the EPDS for men), found much lower optimal cut-off scores than those for English-speaking western women and men. They gave as a possible reason for this finding that “Vietnamese people tend to report somatic symptoms more openly than psychiatric symptoms” (p. 286), and thus an instrument such as the EPDS, which does not include somatic symptoms, will result in a lower cut-off score being required to detect probably depressed people.

Harrington et al. [22] possibly best sum up this view, that one should not simply use a validated cut-off score from one culture with participants from another, or believe that an instrument developed for one culture is unquestionably valid in another culture, stating:

“Researchers and practitioners who use the EPDS (and PHQ-9) should be aware of the tools’ limitations in their context and population … (and with) persons from diverse cultures whose conceptualizations and experiences of depression may not be fully assessed with Western-based screening tools even if validated quantitatively. New or adapted instruments that capture local linguistic and behavioral expressions of depression may need to be developed to improve accuracy of depression screening and diagnosis” (p. 958)

With respect to not just culture, but also the optimal cut-off scores on screening instruments for different perinatal time periods (antenatal/postnatal, or even different trimesters), Lau et al. [23] stated that “a cross-cultural understanding of the different cut-off points during different perinatal periods is crucial …” (p. 1141). They also stated that their findings of different cut-off scores being optimal for women from different areas of China, showed “the importance of proper validation for a psychiatric rating instrument in the different regions of China ” (p. 1149).

### 3.2. Examples of Studies That Could Be Seen to Support the Argument That Screening Scales Do Not Need to Be Validated for Women, and Men, from Different Cultures or Countries

But in contrast to the above, there are an increasing number of studies that, for various reasons, either give the impression that the use of a validated cut-off score for a particular country, cultural group, or gender is not necessary, as a different one will suffice if it has been validated in a different group, or give the impression that one cut-off score could be used for all groups. The reasoning for these views seems to fall into five categories.

#### 3.2.1. Continued Errors in Reading the Literature Regarding the Correct Validated Cutoff Scores

In 2006 I, with colleagues [17], reported on the frequent errors in reporting validated cut-off scores on the EPDS. Unfortunately, such errors continue. Examples where investigators have misinterpreted the original validation study’s cut-off score for English-speaking postnatal women by Cox et al. [1] include those by [5,24,25,26]. In addition, Wroe et al. [27] incorrectly interpreted numerous studies when discussing the choice of the EPDS cut-off score for men, as explained by Matthey [28].

While incorrectly using a cut-off score of just one or two points difference from the validated score may seem to be negligible, Matthey et al. [17] demonstrated that not only can this have a major impact on reported rates of ‘high scorers’, but also—importantly—on the interpretation of whether or not rates of possible depression remain stable from pregnancy to postpartum.

#### 3.2.2. Investigators Use a Validated Cutoff Score from a Different Cultural or Gender Group, Sometimes without Discussing the Possible Pitfalls of Doing This

Examples of such studies include:

Maleki et al. [29], in a study on Iranian fathers, used an EPDS cut-off score of 10 or more based upon that used in a study of principally Portuguese men [30]. They did not however give a rationale for using the same cut-off score for men from these two cultures, nor did they comment on the fact that the Portuguese study that they referred to had not given an empirical rationale for the cut-off score chosen for their men.

Do et al. [31] used a cut-off score of 12 or more for their Vietnamese female participants, without specifying why this was chosen, nor referring to an earlier study that had validated the EPDS for Vietnamese women against depression and some anxiety disorders, with a much lower cut-off score of 4 or more being optimal [4].

Affonso et al. [32] used the one cut-off score (of 10 or more) for women from nine different countries, across five continents. They chose this score as it was recommended by Cox et al. [1] in their original EPDS validation study with English-speaking women, but they did not discuss whether any of the other countries had had validation studies conducted (which in some cases there had been—e.g., Italy and Sweden), nor whether there could be an argument for questioning the use of an Anglo-score for women from very different cultures.

#### 3.2.3. Investigators Choose to Use the Same Cutoff Score for Two or More Groups (e.g., Men and Women) or Different Cultures So That a Comparison Can Be Made Regarding Rates of Possible Depression, Even If They State That the Groups Have Different Validated Cutoff Scores

Examples of such studies include:

Afolabi et al. [16] used 13 or more on the EPDS for each of the three groups of mothers in their study: British mothers in the UK, immigrant Nigerian mothers in the UK, and Nigerian mothers in Nigeria. While they did however discuss how Nigerians can be less inclined to express distress through psychological, as opposed to physical or somatic symptoms, they did not discuss an earlier validation study on Nigerian women and the EPDS which had found a lower score was optimal [7].

Ramchandani et al. [33] used the same EPDS cut-off score for women and men, despite noting that the measure had been validated for men (who had a different optimal cut-off score to that for women). They stated “We therefore used the cut-off of >12 (13+) for both mothers and fathers for comparability” (p. 391).

Gonzalez-Mesa et al. [34] used 13 or more on the EPDS for both their Turkish and Spanish-speaking women, despite the fact that their reference regarding the Spanish version of the EPDS [35] reported that a different cut-off score, of 11 or more was found to be optimal.

Shakeel et al. [36] (2018) conducted a study using the EPDS in several cultural groups in Norway, with participants from Norway, Vietnam, Iran, Turkey, Pakistan, Sri Lanka, Eastern Europe, Africa south of the Sahara, East Asia, and South and Central America. They used a cut-off score of 10 or more for all of these groups, giving the rationale that this had been used in other epidemiolocal studies. While they report cultural differences with respect to perinatal traditions, and how this might be related to the obtained prevalence rates of possible depression, there is no discussion as to whether or not using the same cut-off score for such diverse groups is thus therefore the best approach.

#### 3.2.4. Commonly Used/Internationally Recognized Cutoff Scores

Studies state that a certain cut-off score is now the internationally recognised one, though this seems to suggest that some International perinatal body has decreed this to be so, which to my knowledge is not the case. Those that state that a certain cut-off score is the one most commonly used are however often accurate, though this is usually because of two factors: (a) more studies have been conducted and published in English-speaking populations, which thus use the English-speaking validated EPDS cut-off score; (b) many studies from other cultures then also use these English-speaking validated cut-off sores, either inadvertently, or without fully justifying this, or not pointing out possible limitations of this approach (e.g., see Section 3.2.2 and Section 3.2.3 above).

Examples of such studies include:

Afolabi et al. [16] (2017) state: “… more recent studies have tended towards a general consensus for EPD cut-offs at 13 or more …” (p. 429). Redinger et al. [37] state: “The internationally recognized threshold score for probable depression of ≥ 13 was used” (p. 31); Levis et al. [38] state “… (scores on the on the EPDS) of 10 or higher and 13 or higher (are) typically used to identify women who might be depressed” (p. 1), and Eberhard-Gran et al. [39] state that a score of 10 or more “is frequently used in recent publications” (p. 114). Wesselhoeft et al. [40] used 13 or more for their samples of Danish, Vietnamese, and Tanzanian women, saying this score “is often used to identify women at risk for perinatal depression”(p. 59), citing English-speaking validation studies [1,41].

#### 3.2.5. Meta-Analyses and Systematic Reviews Report Commonly Used Cutoff Scores or Report the Overall Optimal Cutoff Score on the EPDS by Aggregating Data from Different Studies

Examples of such studies include:

Levis et al. [38], using data from 58 studies across multiple cultures, concluded that 11 or more on the EPDS was the optimal screen-positive score when combining its sensitivity and specificity values. While they are clear that they are not recommending that services, or researchers, should simply use this cut-off score regardless of other factors (such as whether a service wishes to maximise sensitivity over specificity), and they are also clear that they were unable to do any cultural sub-group analyses, it is an interesting analysis that could lend weight to the argument that one cut-off score may suffice for all groups, rather than the need to consider different cut-off scores for different cultures.

Of note also is the meta-analysis of studies with men, by Cameron et al. [42]. These authors commented on the number of different EPDS cut-off scores used across the studies, making their analyses problematic. They thus stated “cut-off scores should be standardized (across measures) to ensure continuity in the literature” (p. 199). This difficulty, of using different cut-off scores to compare rates across cultures or groups, was also reported by Woody et al. [43] in their systematic review of perinatal depression in women. These views could be seen as a recommendation that one cut-off score should be agreed upon for use with men or women from all cultures, so as to allow comparisons in prevalence rates.

In addition, meta-analyses and reviews do not usually (if ever) have, as a stated criterion, that studies will only be included in their analyses if they have used the correct validated screening scale cut-off score for their population (or adequately discussed why they have not done so). This means that studies may be included in reviews or meta-analyses that have not used the empirically determined cut-off score for their sample, or not discussed potential limitations of this, thus lending weight to the argument that this is not a particularly important criterion.

## 4. Implementation in Clinical Practice

One could argue that validating a cut-off score is only really useful if is then used within clinical practice. If, however, services decide that it is too impractical to ensure women (or men) from different cultural backgrounds are screened using the validated cut-off score for their culture or gender, then having such validation studies is not clinically useful. Such impracticality could be for many reasons, including staff training difficulties, software modification difficulties, as well as the lack of specific research on when a migrant to a country is acculturated enough to warrant the use of the validated cut-off score on a scale for that country’s ‘indigenous’ population, rather than from their ‘home’ country.

For example, in the health service where I work in Sydney, Australia, one cut-off score—the Anglo validated one (13 or more in pregnancy for possible minor depression)—is used for all women, regardless of culture. Brann et al. [26] also report a similar situation in Sweden. In Denmark, however, while the recent validation study [9] has led to the implementation of the validated Danish cut-off score in their clinical services, a group has been set-up to consider how the health service should implement the most appropriate cut-off score for non-Danish speaking women [44].

## 5. Discussion

Arguments, or implications, that validation studies are not warranted, appear to fall into various categories, as described above (Section 3.2.1, Section 3.2.2, Section 3.2.3, Section 3.2.4 and Section 3.2.5). I would argue that the research in this area would be greatly improved if investigators carefully consider, and justify, their use of screen-positive cut-off scores on whichever scale they are using in each of these categories. This is particularly important given the reports that different cultures or gender may express negative emotions differently (e.g., 4, 9, 10, 12), and hence screening scales need to be validated for each culture or gender group, and that these scores should thus be used, both in research and clinical practice.

Thus, studies that incorrectly cite a validated cut-off score (e.g., those similar to Section 3.2.1) need to be more diligent in their reading of the original source literature. Indeed, it is likely that many such errors are made because the investigators are only reading secondary literature, and they assume that this literature is accurate in the reporting of the primary source material, which unfortunately is often not the case.

If studies use a cut-off score that has not been validated for their population (e.g., those similar to Section 3.2.2), they should clearly state this, and discuss the implications of their decision. Similarly, those that choose to use the same cut-off score between different cultural or gender groups to facilitate comparisons, despite having explained that there are in fact different validated cut-off scores for their groups (e.g., those similar to Section 3.2.3), should discuss the implications of their decision, and how their findings would be different if they had used the validated score for each group.

Those that use a cut-off score based upon it being frequently used, or ‘internationally recognised’ (e.g., those similar to Section 3.2.4), should reference the body that states this, and should discuss the implications of whether or not women, or men, who are distressed may be missed if their group have been shown to require a lower cut-off score.

Finally, those studies that are similar to category Section 3.2.5, where they use aggregating techniques to determine which cut-off score can be used for all groups, should also discuss the implications of this strategy, with respect to the misclassification of the women or men from different cultural groups where their validated cut-off score is different to the one being proposed for all groups.

Journals could improve their evaluation of such studies by requiring authors to state that they have read the source material (and not just secondary material); that they have ascertained if a validated cut-off score exists, or does not exist, for their groups, and to give their rationale as to why they are choosing not to use this/these (if this is the case); and to discuss the implications of their findings if they had instead used the validated cut-off score if there is one (or different cut-off scores if more than one has been validated for that group). In particular, implications need to highlight whether the use of non-validated cut-off scores may therefore misclassify women, or men, as to their emotional health status (screen positive or screen negative).

## 6. Conclusions

There are many studies that determine the optimal screen-positive cut-off score for emotional health screening instruments, such as the EPDS, for women and men from different cultures or countries. These studies show that there is a great range of optimal scores depending upon the variables of culture and gender. There are also however a substantial number of studies which appear to indicate that validating cut-off scores for culture and gender is not considered that important, for a variety of reasons.

Clinical services and researchers need to be mindful of this difference in perspective or approach, and openly discuss this problematic issue when using screening instruments such as the EPDS.

## Data Availability

Not applicable.
